# Impact of Difficult Intravenous Access and Ultrasound-Guided Cannulation Teaching on Healthcare Professionals: A Mixed-Methods, Multi-site Study

**DOI:** 10.7759/cureus.91007

**Published:** 2025-08-26

**Authors:** Krishan Nandapalan, Aidan Smallwood, Kathryn Gething, Hugh Alberti

**Affiliations:** 1 Medical Education, Newcastle University, Newcastle upon Tyne, GBR; 2 Medicine, Newcastle upon Tyne Hospitals National Health Service (NHS) Foundation Trust, Newcastle upon Tyne, GBR; 3 Anesthetics, Newcastle upon Tyne Hospitals National Health Service (NHS) Foundation Trust, Newcastle upon Tyne, GBR; 4 Psychology, Newcastle upon Tyne Hospitals National Health Service (NHS) Foundation Trust, Newcastle upon Tyne, GBR

**Keywords:** emotional impact, intravenous cannulation, postgraduate medical education, simulation trainer, ultrasound-guided vascular access

## Abstract

This was a mixed-methods study designed to evaluate the impact that difficult intravenous access situations and ultrasound-guided cannulation teaching have on healthcare professionals (HCPs). Quantitative data were collected from pre- and post-surveys from a multi-site ultrasound-guided cannulation course (n = 252). The difference in self-reported confidence between the surveys was analysed for significance using a complete case analysis, a two-tailed paired samples T-test with a 95% confidence interval. Qualitative data were collected from two semi-structured focus groups with a total of 13 participants and analysed using reflexive thematic analysis. Difficult cannulation situations negatively impact HCPs, causing workflow disruptions and emotions such as guilt, helplessness, and self-doubt. After receiving ultrasound-guided cannulation teaching, course participants felt significantly more confident in their ability to cannulate patients (t = -9.55, p < 0.001), identify peripheral structures using ultrasound (t = -20.51, p < 0.001), and use ultrasound to aid cannulation (t = -32.93, p < 0.001). Focus group participants felt that ultrasound-guided cannulation teaching reduced the negative impact of subsequent difficult cannulation experiences.

## Introduction

Peripheral intravenous cannulation (PIVC) to establish intravenous (IV) access is the most common invasive procedure in hospitals, allowing for blood samples to be taken for laboratory tests and interventions to be delivered intravenously [1]. In England, PIVC is an essential skill for doctors and is becoming increasingly important for other professions such as nurses and radiographers [2,3].

Difficult intravenous access

'Difficult intravenous access' (DIVA) is a term typically used to describe situations in which gaining IV access requires multiple PIVC attempts, for example, due to challenging anatomy. A systematic review found that DIVA is a well-documented phenomenon; however, reported prevalence varies dramatically due to varying definitions. The authors proposed that DIVA be defined as 'when a clinician has two or more failed attempts at PIVC using traditional techniques, physical examination findings are suggestive of DIVA (e.g. no visible or palpable veins), or the patient has a stated or documented history of DIVA' [4].

DIVA can lead to patients undergoing multiple PIVC attempts that are painful, risk iatrogenic harm, and delay investigations and treatment [5]. Anecdotally, DIVA negatively impacts the healthcare professionals (HCPs) attempting PIVC; however, this is poorly characterised in the literature, often being omitted or described vaguely with minimal evidence [6].

Ultrasound-guided peripheral intravenous cannulation

Often, the solution for DIVA is escalation to trained specialists who perform PIVC more frequently and with greater operator proficiency, such as anaesthetists. However, this role is auxiliary to the group's primary function, and the burden of DIVA has led some sites to establish dedicated vascular access teams [7,8]. Specialists are often adept at ultrasound-guided peripheral intravenous cannulation (US-PIVC). Compared to the traditional landmark approach, US-PIVC has a significantly higher success rate, requiring fewer attempts, especially in DIVA [9,10]. However, non-specialists often have limited experience with ultrasound, as it is uncommon in the undergraduate curriculum and not a requirement for new graduates. Therefore, there is a growing demand to train more HCPs in US-PIVC to decrease the number of failed attempts and subsequent escalations [11,12].

Multiple studies have demonstrated that US-PIVC can be effectively taught to nurses, doctors, and medical students with limited prior ultrasound experience in a single teaching session, leading to greater procedural confidence, knowledge, and competence [13]. However, they rarely extend to other members of the multidisciplinary team, such as advanced clinical practitioners, radiographers, and vascular access practitioners. Furthermore, they are often single-site studies, have limited participant numbers, and are conducted outside the United Kingdom. Therefore, some findings may not be transferable due to differences in international job roles, demographics, and training curricula.

This study aimed to answer two research questions: (1) How does the experience of difficult IV cannulation affect HCPs? (2) How do HCPs benefit from ultrasound-guided cannulation teaching?

## Materials and methods

This was a convergent parallel mixed-methods study with quantitative survey data collected from a US-PIVC course and qualitative data from two focus groups. Ethical approval was granted by the Research Ethics Committee of Newcastle University (approval number: 36214/2023). We obtained informed consent from all participants, who were free to withdraw participation at any time.

UltraCann course

In August 2023, we established a half-day US-PIVC course across the North East and North Cumbria Integrated Care Board (NENC ICB). The course was named ‘UltraCann’ and delivered via the non-profit organisation ‘A-LiNE’ (Anaesthetic Learning in the North East). The course comprised approximately 30 minutes of lectures about theory and technique, 45 minutes of participants scanning peripheral veins on each other, and 60 minutes of participants performing US-PIVC on homemade phantoms.

UltraCann was open to all HCPs within the NENC ICB, and a baseline of prior PIVC experience was advised. Participants could book the course using A-LiNE’s website or, at some sites, through their hospital’s education department. UltraCann was initially free to attend, but the cost was increased to £20 during the study to cover consumables and reduce the number of participants who booked but did not attend. Each course had up to 10 participants with two or three teachers who were predominantly anaesthetic registrars and consultants. Each site had five Butterfly iQ ultrasound probes (Butterfly Network, Inc., Burlington, MA, USA) to be used with an Apple 9th Generation iPad (Apple Inc., Cupertino, CA, USA), funded by a Health Education England grant. Consumables included 18G IV cannulas, homemade US-PIVC phantoms, ultrasound gel, disposable gloves, and paper towels.

Quantitative data

We asked all UltraCann course participants from 1st October 2023 to 30th September 2024 (inclusive) to complete pre- and post-course surveys. All questions presented in this study were seven-point Likert scales designed following ‘Developing questionnaires for educational research: AMEE Guide No. 87’ [14]. The key metrics of interest across the surveys were self-reported confidence in PIVC, use of ultrasound, and performance of ultrasound-guided cannulation. The pre-course survey also contained questions on participant demographics and experiences with DIVA. The post-course also contained questions on perceived interest, importance, and satisfaction regarding the course. The surveys were designed with consideration of the four levels of the Kirkpatrick Evaluation Model [15]; thus, this was also used to evaluate the outcomes.

We analysed the difference in self-reported confidence between the course surveys for statistical significance using a complete case analysis, a two-tailed paired samples T-test with a 95% confidence interval. For the analysis, we assigned the Likert responses numerical values (completely unconfident = 1, completely confident = 7) and measured the effect size using Cohen’s d. We analysed all data using SPSS Statistics version 29.0 (IBM Corp. Released 2022. IBM SPSS Statistics for Windows, Version 29.0. Armonk, NY: IBM Corp.).

Qualitative data

We conducted two independent, semi-structured focus groups virtually on Microsoft Teams (Microsoft Corp., Redmond, WA, USA) with two members of the research team: a principal clinical psychologist and a specialised foundation programme doctor. Each focus group was scheduled for a maximum of two hours. Participants were eligible to join the focus group if they were at least 18 years old, HCPs who performed PIVC as part of their clinical role, and employed by a trust within the NENC ICB. Additionally, participants for the first focus group must not have attended UltraCann, and participants for the second focus group must have attended UltraCann within the last year. We recruited participants via hospital education departments, word of mouth, and email.

The question schedule for the first focus group concentrated on experiences with DIVA, whereas the second group concentrated on experiences with US-PIVC. The conversations were recorded and transcribed using Microsoft Teams’ built-in function. The transcripts were manually verified and corrected against the recording by the research team before pseudonymising the transcripts and deleting the recordings. The transcripts were analysed following Braun and Clarke’s six-phase approach to reflexive thematic analysis [16]. We used a bottom-up, open-coding approach to generate initial codes. The codes were then refined and grouped into sub-themes and major themes that related to our research questions.

## Results

Surveys

Between 1st October 2023 and 30th September 2024, 280 UltraCann attendees completed a course survey. Of these, six only filled out the pre-survey, 21 only filled out the post-survey, and one did not consent to the use of their responses in research.

Demographics

**Figure 1:**
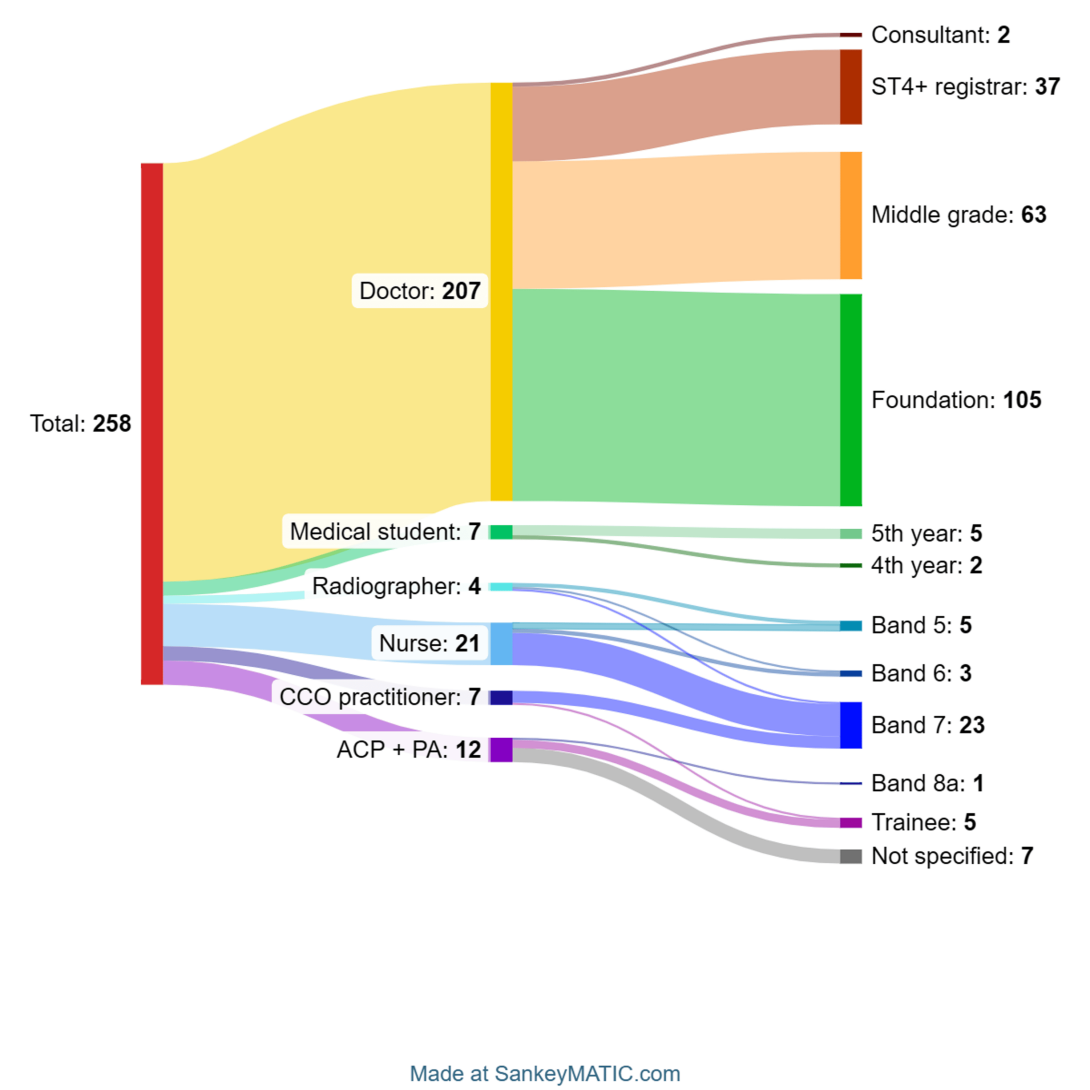
Course participant breakdown by job role then grade (n = 258) ST4+: specialty trainee year 4 and above, CCO: critical care outreach, ACP: advanced clinical practitioner, PA: physician associate

We collected demographic information on the pre-course survey (n = 258), overall spanning seven hospitals in six trusts within the NENC ICB. The majority (80.5%) of participants were doctors (Figure 1). For simplicity, advanced clinical practitioners and physician associates were grouped, and doctor grades were grouped into foundation doctor (years 1 and 2), middle grade doctor (anyone who had completed foundation years but was working below the level of an ST4 registrar), registrars working at the level of ST4 or higher, and consultants.

Experiences With Difficult Cannulation

**Figure 2:**
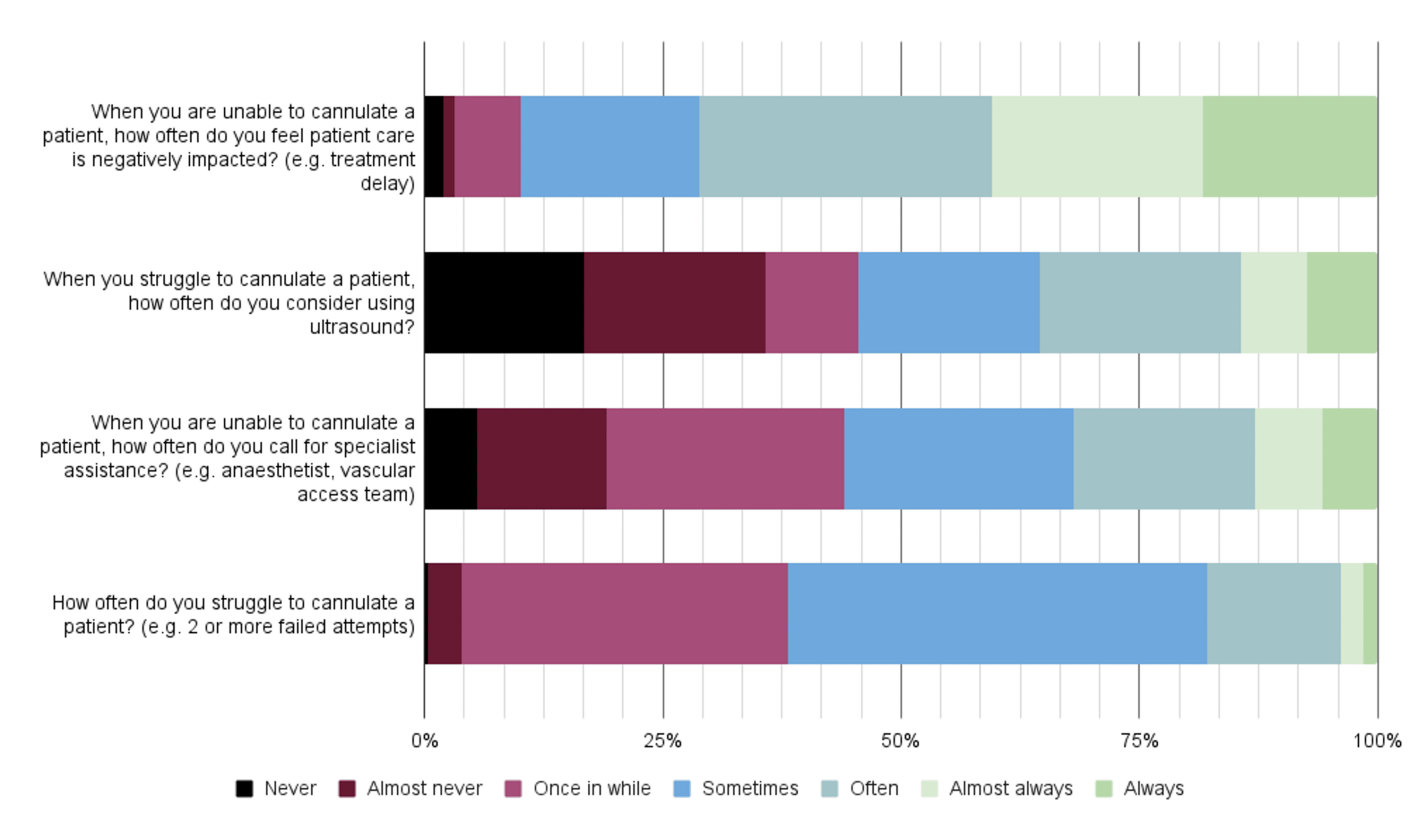
Course survey responses to questions relating to difficult cannulation experiences (n = 258) The data are presented as the number of responses to each question.

We collected data about difficult cannulation experiences on the pre-course survey (n = 258). Most participants struggled to cannulate patients' sometimes' (median, mode) (Figure 2). In this situation, participants considered using ultrasound 'sometimes' (median) or 'often' (mode), with 43 participants never considering it. When participants were unable to cannulate a patient, they 'often' (median, mode) felt patient care was negatively impacted and called for specialist assistance, such as an anaesthetist or vascular access team, 'once in a while' (mode) or 'sometimes' (median).

Changes in Confidence

**Figure 3:**
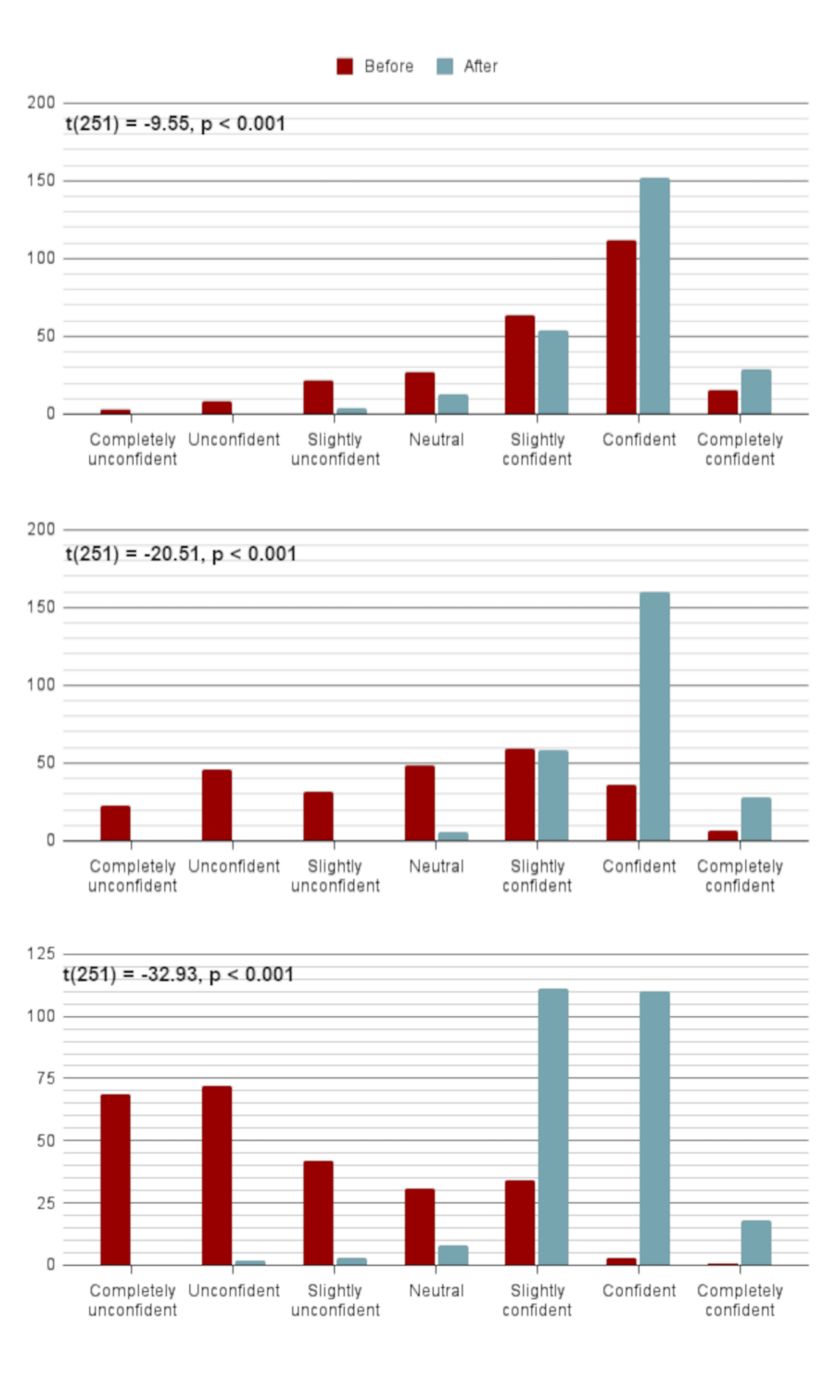
Self-reported confidence before and after the UltraCann course (n = 252) The data has been presented as the number of responses to each question. The difference in self-reported confidence before and after the course was analysed for statistical significance using a complete case analysis, a two-tailed paired samples T-test with a 95% confidence interval. (a, top) How confident are you in your ability to cannulate patients? t(251) = -9.55, p < 0.001. (b, middle) How confident are you in identifying peripheral structures (e.g. veins, arteries, nerves) using ultrasound? t(251) = -20.51, p < 0.001. (c, bottom) How confident are you in using ultrasound to aid cannulation? t(251) = -32.93, p < 0.001.

We collected confidence data on both the pre- and post-surveys, but only responses from participants who completed both surveys were analysed (n = 252). The results indicated that there was a significant increase in participants' confidence in their ability to cannulate patients before (M = 5.15, SD = 1.27) and after (M = 5.75, SD = 0.79) the course ( t(251) = -9.55, p < 0.001. Cohen's d = 0.60. (Figure 3a)); identify peripheral structures using ultrasound before (M = 3.84, SD = 1.65) and after (M = 5.83, SD = 0.64) the course (t(251) = -20.51, p < 0.001. Cohen's d = 1.29. (Figure 3b)); and use ultrasound to aid cannulation before (M = 2.61, SD = 1.44) and after (M = 5.50, SD = 0.79) the course (t(251) = -32.93, p < 0.001. Cohen's d = 2.29. (Figure 3c)).

Interest, Importance, and Content

We collected data regarding how interesting, important, and satisfying participants found the teaching on the post-course survey (n = 273). Participants were 'very satisfied' (median, mode) with the content of the course and perceived it to be 'very interesting' (median, mode) and 'very important' (median, mode).

Focus groups

Demographics

The first focus group comprised seven participants who worked as foundation doctors across five hospitals in three trusts. The second focus group comprised five participants: two foundation doctors, a middle-grade doctor, a band seven nurse practitioner, and a physician associate, who worked across three hospitals in two trusts. One foundation doctor was a participant in both focus groups.

Themes

Four major themes were identified from the focus group transcripts: challenges of DIVA, emotions relating to difficult cannulation experiences, the impact of US-PIVC in DIVA, and the challenges of using ultrasound in clinical practice. The themes are presented with a selection of representative quotes in Table 1.

**Table 1:** Major themes and representative quotes The focus group transcripts were analysed using a bottom-up, open coding approach to generate initial codes. The codes were then refined and grouped into sub-themes and major themes that related to our research questions. DIVA: difficult intravenous access

Theme	Representative quotes
Challenges of DIVA	‘Something that I find very tricky is when someone is about to go for an urgent scan and the porter rocks up and you've just been told they need a cannula, and then you have to cannulate under pressure and the porter's cross with you, radiologists cross, the patients cross. They've got this sort of pressure.’
	‘It's so dependent on the patient and I don't know how to predict how people are going to take it. Sometimes people prefer being distracted, some appreciate you being empathetic and others are just very annoyed by whatever I do.’
	‘It just kind of ruins your planning cause you assume it's gonna take minutes, but you know you might be there 30 minutes and then you might need to call someone. And then when someone comes, you need to be with them as sort of a courtesy as well. So then it could easily take an hour. So then that sort of throws the whole plan outside the window.’
	‘I think sometimes the chain of command is bureaucracy rather than actual skill and experience.’
	‘You've got a patient telling you “you will need anaesthetics, even they struggle with me”. I completely see why they don't want me to try, but I'm sort of telling them like the first on call anaesthetist isn't going to want to know if I don't at least try. It's just a procedural thing that you have to jump through.’
Emotions relating to difficult cannulation experiences	‘It's your job as an F1 to cannulate people. When you fail, you feel like you're not doing your job.’
	‘It feels like quite a silly thing to be upset about. I think I'd feel quite embarrassed saying to someone I'm really upset because of this cannula. It always seems like such a routine thing. I wouldn't necessarily want to talk to people about it.’
	‘When you asked if it's emotional, I initially thought no, not really. But then I realised that my biggest highs were after getting a difficult cannula in and then my lowest low was probably after failing a few cannulas.’
	‘Calling anaesthetists when you know the emergencies they're probably dealing with to help you with a cannula feels quite trivial and you feel quite guilty asking them. Like I know this isn't your job, but there's no one else.’
	‘The registrar said. Well, you're far more experienced than I am because I've not done them in five years.’
	‘I called anaesthetics and they said, “It's not my job to run around the hospital doing cannulas for the junior doctors.” And then I was really lost with where to go with that.’
	‘You're not going to want to call that person when they've made you feel really stupid.’
	‘It's more the effect that it has on the patient and the effect that their emotions have on you.’
	‘Patients say, “You can give it a go, but I'm not happy about it.” And you think, “I'm trying to help you and I appreciate it's difficult. Just feel a bit sad about this now.”’
	‘I'll give it one more go and then I'll give you a little break. Which means I want to give myself a little break. Because I'm about to have a breakdown.’
Impact of ultrasound	‘We'll tackle the harder ones now because I know that if I can't get it, I can have a go with the ultrasound.’
	‘There's been a lower threshold of coming to grab me or someone else who’s done the course to go and get the ultrasound rather than having another few goes.’
	‘I've stabbed people less. I've caused less pain. You know, do no harm.’
	‘As soon as you get the machine out, if there's juniors on your ward, they expect to be trained, which is fine. You do end up feeling like you're getting watched by everyone.’
	‘Sometimes when you fail with ultrasound you can kind of open the gateway to anaesthetics.’
Challenges of ultrasound	‘It's just finding a machine asking, begging, borrowing and nobody wants to lend you it.’
	‘You are doing landmark cannulas so quickly, so one of the things that we have to learn with a probe is to slow down and that is so hard.’
	‘Like working out, hold it in one hand, cannulate with the other.’
	‘I've literally been given all the tools to succeed, and I haven't.’
	‘From a patient point of view, their expectations of you getting it in with ultrasound are 100% higher. But when you fail at ultrasound, it's like the end of the world, isn't it for you? And the patient’s going, “Well, they normally get it in” and then you think, “ohh it's me”.

## Discussion

How does the experience of difficult cannulation affect healthcare professionals?

Our novel study has confirmed that DIVA can have a profound impact on the workflow and emotions of HCPs. We know that PIVC is the most common invasive procedure in hospitals [1], and most course participants struggled to cannulate patients 'sometimes', so any HCP who is expected to cannulate will inevitably experience DIVA. These situations are variable and require an unpredictable amount of time with no guarantee of success. This can compound the already busy workload of HCPs and can lead to investigation and treatment delays for patients [6,9,13]. This was echoed by course participants ‘often’ feeling patient care was negatively impacted when they were unable to cannulate a patient.

Course participants had to call for specialist assistance ‘once in a while’ (mode) or 'sometimes' (median), indicating that escalations outside of the immediate team are not uncommon. Each step up the chain often correlates with less availability; for example, more senior doctors or anaesthetists may be busy in the clinic, theatre, or managing emergencies. Furthermore, each escalation results in more PIVC attempts for the patient and longer time delays. HCPs feel obligated to attempt cannulation before escalating, even if they cannot see or palpate any veins. This can feel like a futile exercise that causes unnecessary pain, leading HCPs to become frustrated and resent the de facto protocols that are in place. Furthermore, some patients lose confidence in HCPs after failed attempts or if they have required escalation in the past and may request a more senior HCP to cannulate them, which reinforces the self-doubt that HCPs experience. This can lead to difficult patient conversations where HCPs must defend their decision to attempt PIVC before escalation, as they feel bound to a protocol that they resent.

DIVA situations can therefore provoke a range of emotions in HCPs that may relate to patients, other staff, or solely themselves. Many of these emotions stem from the sentiment that PIVC is a straightforward task and the sole responsibility of the HCP, which led to some participants subconsciously minimising the impact it had on them. It became apparent that there is an inescapable sense of guilt and self-doubt for HCPs from every outcome of DIVA bar success: repeated failed attempts, treatment delays, or having to escalate. Furthermore, DIVA situations can cause challenging conversations between HCPs and other staff or patients, causing feelings of frustration, helplessness, and sadness. These emotions are only intensified by the pressure of the situation: a jeopardised rapport with a fed-up patient following a difficult conversation, an apathetic anaesthetist on the other end of a phone, an audience of staff and visitors expectantly waiting for your success, and a growing list of jobs and missed calls, all while an HCP apologises again and unsheathes their next cannula.

How do HCPs benefit from ultrasound-guided cannulation teaching?

The benefits of the course were considered in line with the four levels of the Kirkpatrick Evaluation Model [15]. Firstly, participants were 'very satisfied' with the course and perceived it to be both 'very important' and 'very interesting', providing evidence for level 1 (reaction). Participants were significantly more confident in their ability to cannulate, use ultrasound, and perform US-PIVC, providing evidence for level 2 (learning). The increased confidence in landmark PIVC had a medium effect size. It may be due to participants being able to visualise venous anatomy under ultrasound, which can give participants a better understanding of why their attempts may fail. The increased confidence in the use of ultrasound for identifying peripheral structures and guiding PIVC had large and very large effect sizes, respectively. Increased confidence for participants from a range of job roles and grades is therefore a clear benefit of the course, and these findings are corroborated by similar US-PIVC course studies [11-13,17].

Focus group participants described their newly acquired US-PIVC skill as straightforward to implement in clinical practice, providing evidence for level 3 (behaviour). However, there was sampling bias for the second focus group, as participants were unlikely to have volunteered if they had not attempted US-PIVC after the course. One study found that less than half of respondents had attempted US-PIVC following the course [12]; however, this may be confounded by barriers such as ultrasound availability. This was the largest perceived barrier in our study, with access varying dramatically by hospital, department, and time of day. When readily available on the ward, they were utilised frequently, and HCPs had a much lower threshold for performing US-PIVC. Conversely, when they were scarcely available, HCPs considered US-PIVC a last resort, knowing it would take a lot of time and effort to find an ultrasound, cannulate, and then return it. This led to some HCPs underutilising their US-PIVC skills and feeling frustrated about the lack of availability.

Participants who had performed US-PIVC felt that it conveyed fewer individual attempts and less escalation, which were common causes of grievance with patients and seniors. They also felt less guilty about causing unnecessary pain, knowing they had taken steps to maximise their chance of success. Furthermore, if participants had attempted using ultrasound, they felt more comfortable escalating to specialists, as they were reassured that the anatomy was sufficiently difficult to warrant expertise. Additionally, the ability to perform US-PIVC allowed HCPs to pass on their knowledge, which was a source of pride for some and stress for others. Therefore, US-PIVC ability reduced the frequency of events that triggered negative emotions in HCPs and provided new opportunities that generated positive emotions, providing evidence for level 4 (results). However, the expectation of success when performing US-PIVC rather than landmark PIVC was notably higher among patients, HCPs, and observers, which could make the situation more stressful and failure more embarrassing. The potential benefits likely outweigh this, but it is important to acknowledge that the emotional impact of US-PIVC runs in both directions.

Strengths and limitations

The focus group sample sizes were sufficient to establish a greater understanding of the ways difficult cannulation and US-PIVC teaching affect HCPs. However, it became clear that their job role and location influenced an individual's perspective and behaviour. For example, some participants were from trusts that had nurses who routinely cannulated and specialist vascular access teams, which changed how participants escalated cannulas. Therefore, repeating the focus group with staff from different job roles, at different grades, and from different trusts may yield new perspectives that were not uncovered in this study.

Overall, the data provided some evidence for how HCPs directly benefited from ultrasound-guided cannulation teaching. However, there were consequential impacts on other groups: some HCPs were able to be taught US-PIVC by HCPs that attended UltraCann, specialists likely received fewer PIVC escalations, and patients likely suffered fewer failed attempts and treatment delays as a result. These impacts were not measured in this study. However, we implore anyone reproducing this study to consider them, for example, by conducting patient surveys and measuring the volume of escalation calls before and after implementation of the teaching. This information would facilitate a more holistic evaluation of the impact of US-PIVC teaching.

Implications

Firstly, we encourage readers to reflect on the notion that cannulation situations can have profound emotional impacts on HCPs, even though it is seldom discussed. US-PIVC can make these experiences more positive, thereby benefiting HCPs in a way that may not be commonly recognised. US-PIVC has clear, well-documented benefits to patients in the form of fewer failed attempts and shorter treatment delays [6,9,13]. US-PIVC can be reliably taught in a half-day session to HCPs from a range of jobs at different grades in the United Kingdom, as evidenced by this study and others [11,13,17]. Therefore, this study provides further justification for the establishment of US-PIVC courses across the United Kingdom due to the numerous resulting benefits.

## Conclusions

PIVC is the most common invasive procedure in hospitals and is widely considered to be a basic skill. However, PIVC can be variable, and DIVA can lead to multiple failed attempts, a disproportionate amount of time spent, and treatment delays before being escalated. For HCPs, this presents many circumstances relating to other staff, patients, or solely themselves that provoke overwhelmingly negative emotions such as guilt, helplessness, and self-doubt. Our half-day US-PIVC teaching session led to participants feeling significantly more confident in their ability to cannulate patients, identify peripheral structures using ultrasound, and use ultrasound to aid cannulation. Our focus group participants felt that US-PIVC conveyed fewer failed attempts, shorter treatment delays, and fewer escalations, thereby reducing the negative emotional responses provoked. For some, it transformed DIVA situations into positive experiences, such as ad hoc teaching sessions; however, for others, it raised expectations and made the situation more stressful. Overall, we strongly advocate for the provision of adequate training and ultrasound machine availability to make US-PIVC more commonplace. Our future work will seek to elaborate on the impact of US-PIVC teaching on HCPs, patients, and services, as well as the cost-effectiveness of providing teaching and ultrasound machines.

## References

[1] Alexandrou E, Ray-Barruel G, Carr PJ (2015). International prevalence of the use of peripheral intravenous catheters. J Hosp Med.

[2] Keenan LY, Muir C, Cuthbertson LM (2001). Maximizing the benefit--minimizing the risk: the developing role of radiographers in performing intravenous injections. Br J Radiol.

[3] Nursing and Midwifery Council (2018). Nursing and Midwifery Council. Future nurse: Standards of Proficiency for Registered Nurses [Internet]. Nursing and Midwifery Council. Standards of Proficiency for Registered Nurses.

[4] Bahl A, Johnson S, Alsbrooks K, Mares A, Gala S, Hoerauf K (2021). Defining difficult intravenous access (DIVA): a systematic review. J Vasc Access.

[5] Witting MD (2012). IV access difficulty: incidence and delays in an urban emergency department. J Emerg Med.

[6] Ng M, Mark LK, Fatimah L (2022). Management of difficult intravenous access: a qualitative review. World J Emerg Med.

[7] Whalen M, Maliszewski B, Baptiste DL (2017). Establishing a dedicated difficult vascular access team in the emergency department: a needs assessment. J Infus Nurs.

[8] Carr PJ, Higgins NS, Cooke ML, Mihala G, Rickard CM (2018). Vascular access specialist teams for device insertion and prevention of failure. Cochrane Database Syst Rev.

[9] van Loon FH, Buise MP, Claassen JJ, Dierick-van Daele AT, Bouwman AR (2018). Comparison of ultrasound guidance with palpation and direct visualisation for peripheral vein cannulation in adult patients: a systematic review and meta-analysis. Br J Anaesth.

[10] Tran QK, Fairchild M, Yardi I, Mirda D, Markin K, Pourmand A (2021). Efficacy of ultrasound-guided peripheral intravenous cannulation versus standard of care: a systematic review and meta-analysis. Ultrasound Med Biol.

[11] Armson AM, Moynihan R, Stafford N, Jacobs C (2021). Ultrasound-guided cannulation for medical students. Clin Teach.

[12] Archer-Jones A, Sweeny A, Schults JA, Rickard CM, Johnson L, Gunter A, Watkins S (2025). Evaluating an ultrasound-guided peripheral intravenous cannulation training program for emergency clinicians: an Australian perspective. Australas Emerg Care.

[13] Hoskins MJ, Nolan BC, Evans KL, Phillips B (2023). Educating health professionals in ultrasound guided peripheral intravenous cannulation: a systematic review of teaching methods, competence assessment, and patient outcomes. Medicine (Baltimore).

[14] Artino AR Jr, La Rochelle JS, Dezee KJ, Gehlbach H (2014). Developing questionnaires for educational research: AMEE Guide No. 87. Med Teach.

[15] Kirkpatrick JD, Kirkpatrick WK (2016). Kirkpatrick's Four Levels of Training Evaluation.

[16] Braun V, Clarke V, Hayfield N, Terry G (2019). Handbook of Research Methods in Health Social Sciences. Handbook of Research Methods in Health Social Sciences [Internet.

[17] Pham M, Aldous R, Brincat S (2024). Implementation of ultrasound-guided cannulation training for foundation doctors. Clin Med (Lond).

